# Pre-conceptional and Peri-Gestational Maternal Binge Alcohol Drinking Produces Inheritance of Mood Disturbances and Alcohol Vulnerability in the Adolescent Offspring

**DOI:** 10.3389/fpsyt.2018.00150

**Published:** 2018-04-23

**Authors:** Anna Brancato, Valentina Castelli, Angela Cavallaro, Gianluca Lavanco, Fulvio Plescia, Carla Cannizzaro

**Affiliations:** Department of Sciences for Health Promotion and Mother and Child Care “Giuseppe D'Alessandro”, University of Palermo, Palermo, Italy

**Keywords:** alcohol, binge drinking, female, perinatal, adolescence, abuse vulnerability

## Abstract

Although binge drinking is on the rise in women of reproductive age and during pregnancy, the consequences in the offspring, in particular the inheritance of alcohol-related mood disturbances and alcohol abuse vulnerability, are still poorly investigated. In this study, we modeled both Habitual- and Binge Alcohol Drinking (HAD and BAD) in female rats by employing a two-bottle choice paradigm, with 20% alcohol and water. The exposure started 12 weeks before pregnancy and continued during gestation and lactation. The consequences induced by the two alcohol drinking patterns in female rats were assessed before conception in terms of behavioral reactivity, anxiety- and depressive-like behavior. Afterwards, from adolescence to young-adulthood, male offspring was assessed for behavioral phenotype and alcohol abuse vulnerability. At pre-conceptional time BAD female rats showed higher mean alcohol intake and preference than HAD group; differences in drinking trajectories were attenuated during pregnancy and lactation. Pre-conceptional BAD induced a prevalent depressive/anhedonic-like behavior in female rats, rather than an increase in anxiety-like behavior, as observed in HAD rats. In the adolescent offspring, peri-gestational BAD did not affect behavioral reactivity in the open field and anxiety-like behavior in the elevated plus maze. Rather, BAD dams offspring displayed higher despair-behavior and lower social interaction with respect to control- and HAD dams progeny. Notably, only binge drinking exposure increased offspring vulnerability to alcohol abuse and relapse following forced abstinence. This is the first report showing that binge-like alcohol consumption from pre-conceptional until weaning induces relevant consequences in the affective phenotype of both the mothers and the offspring, and that such effects include heightened alcohol abuse vulnerability in the offspring. These findings highlight the need for more incisive public education campaigns about detrimental consequences of peri-gestational alcohol exposure.

## Introduction

Despite the increased awareness about the teratogenic effect of *in utero* exposure to psychotropic drugs [1–7], prenatal alcohol exposure represents one of the most common early brain insults [8–10]. Globally, about 10% of women consume alcohol during pregnancy [11], between 5 and 10% admit binge drinking incidents [12] and between 1 and 2% deliver a child with fetal alcohol spectrum disorders (FASD), whose lifelong consequences can range from minor to severe disabilities [13]. Notably, binge drinking (i.e., four to five or more drinks per occasion) prior to pregnancy represents a relevant predictor of perinatal alcohol exposure that might put the development of the offspring in jeopardy [14].

Although several studies have investigated the clinical consequences of alcohol abuse as well as the incidence of FASD, very few is known about the impact of binge alcohol drinking (BAD) on the affective and emotional pattern of females of reproductive age [15].

This is an important issue since modifications in the affective and emotional response of the mothers, as a consequence of long-term alcohol intake, can be inherited by the progeny [16, 17].

Rodent models of alcohol exposure, which can mimic both habitual- and binge human drinking patterns, allow the investigation of the epigenetic mechanisms that underlie dysfunctional emotional and affective behavior, as well as heritable stress-related phenotypes [18–23]. In this regard, well-established behavioral paradigms are essential tools to search specific endophenotypes and provide cues to investigate the neuropathology of mood disturbances. For instance, the exploratory behavior of novel and challenging environments such as the open field and the elevated plus maze, can provide a reliable readout of unconditioned anxiety-like behavior [24–26]; in addition, the animal unconditioned preference for natural rewards, such as sweet solution [27] or social interaction toward a conspecific [28], can be altered in anhedonic-like states, such as depression; lastly, despair behavior in the forced swim test is a largely employed measure of depression-like behavior and has been traditionally used to detect antidepressant properties of drugs [29].

Thus, the first aim of this research was to model pre-conceptional habitual- and binge-like alcohol consumption in female rats, in order to investigate the consequences on their emotional and affective behavior. We employed a long-term, two-bottle choice paradigm with continuous or intermittent access to 20% alcohol and water, and tested the animals for: drinking trajectories, behavioral reactivity, reward sensitivity, anxiety- and depression-like behavior.

It has been reported that mothers who drink before pregnancy often indulge in alcohol consumption during pregnancy and while breastfeeding, transferring alcohol to infants through the maternal milk [30]. Thus, in the present study, female rats continued habitual and binge-like alcohol self-administration along pregnancy and lactation. Notably, the duration of pre- and postnatal alcohol treatment allows to model human fetal alcohol exposure along the whole gestational period, since the first two postnatal weeks in rodents approximately correspond to the last gestational trimester in humans [31, 32].

In spite of the well-established association between *in utero* alcohol exposure and psychiatric risk in the progeny, experience suggests that mental health practitioners infrequently identify FASD before adolescence [33]. The Seattle longitudinal study revealed that 21-year young adults whose mothers reported a binge pattern of alcohol use, displayed significantly higher overall levels of psychiatric symptoms, than those exposed to low or moderate levels of alcohol *in utero* [34]. Adolescents' most substantial deficits, according to the Vineland Adaptive Behavior Scale [35] lied in the socialization domain, reflecting both social anxiety and social avoidance. A similar prospective study highlighted the association between maternal alcohol use during pregnancy and offspring early drinking, suggesting genetic inheritance of alcohol abuse behavior [36–38].

Based on this evidence, we investigated the occurrence of specific variations in the affective and emotional phenotypes of the offspring during adolescence, as a consequence of the perinatal exposure to the two distinct alcohol drinking paradigms. The offspring was tested for social interaction, which explores social reward and embarrassment that may occur in novel social environments [39, 40]. Eventually, the occurrence of a vulnerability to alcohol abuse was investigated by exposing the adolescent offspring to the two-bottle choice paradigm until early adulthood.

To our knowledge, this is the first report on the affective and emotional consequences of long-term binge- alcohol drinking by voluntary self-administration in female rats, and the inheritance of mood disturbances and alcohol vulnerability in the adolescent progeny.

This evidence strengthens the translational value of binge-like alcohol self-administration by the two-bottle choice procedure, in search of mechanisms of multigenerational inheritance of alcohol detrimental consequences [41].

## Materials and methods

### Animals and housing conditions

Adult female nulliparous Wistar rats (200–220 g, Harlan, Udine, Italy) were housed individually in standard rat cages (40 × 60 cm, 20 cm in height), with *ad libitum* access to water and food, in a temperature- (22 ± 2°C) and humidity- (55 ± 5%) controlled room, on 12 h inverted light/dark cycle (08:00–20:00). Rats were gently handled for 3 min per day for a week before the experimental procedures, when they were randomly assigned to one of the three experimental groups, according to the self-administration paradigm: habitual alcohol drinking (HAD), BAD, water drinking controls (CTR).

At the end of the behavioral assessments, each female rat was housed with a breeder male rat.

On gestational day 1 (GD1; i.e., the day when pregnancy was confirmed) eight female rats randomly selected from each experimental group and housed in standard maternity cages, filled with wood shavings. Alcohol consumption was not measured when the male rat was present in the cage [42]. Female rats underwent the self-administration procedure throughout pregnancy, accordingly to the respective two-bottle choice paradigm. Dams were inspected twice daily for delivery until the day of parturition, considered as postnatal day 0 (PND 0); dams and litters were kept in a nursery (24 ± 2°C) and not separated until weaning, in order to avoid confounding effects due to cross-fostering procedure [43–45] and model the human condition.

One male rat from each alcohol drinking- and control litter was randomly chosen to undergo behavioral testing, beginning on PND 23, so that experimental groups of rat offspring were: perinatally habitual alcohol-exposed rats (p-HAD, *n* = 8), perinatally binge alcohol-exposed rats (p-BAD, *n* = 8), perinatally water-exposed controls (p-CTR, *n* = 8).

All the experiments were approved by the Committee for the Protection and Use of Animals of the University of Palermo, in accordance with the current Italian legislation on animal experimentation (D.L. 116/92) and the European directives (2010/63/EU).

### Two-bottle “20% alcohol vs. water” choice drinking paradigms

Female rats underwent home cage two-bottle “alcohol vs. water” choice regimen, according to the experimental group assigned. Indeed, HAD rats were given 24 h free choice between one bottle of alcohol (20% v/v) and one of tap water, 7 days per week, except during the behavioral tests (weeks 8–11) when they were given 5 drinking sessions per week; BAD rats were given 24 h alcohol (20% v/v) access during 3 sessions per week, i.e., on Monday, Wednesday, and Friday, while they received 2 bottles of tap water on the intervening days. CTR rats received two bottles of water. Overall, HAD rats underwent 76 pre-conceptional-, 21 gestational, and 21 post-gestational drinking sessions. On the other hand, BAD rats had 36 pre-conceptional-, 9 gestational, and 9 post-gestational drinking sessions.

Alcoholic solution (20% v/v) was daily prepared by diluting alcohol 96° (Carlo Erba Reagenti, Italy) with tap water. Plastic bottles (120 ml; metal cap 0.8 mm diameter hole, Tecniplast, Italy) were filled every day with 100 ml of freshly prepared solution, in alternative left-right position, to avoid side preference, and presented at lights off. Alcohol- and water intake were measured 1 h after lights off and the day after, immediately before lights-off, by weighing the bottles. Possible, fluid spillage was monitored by using multiple bottles filled with water and alcohol 20%, allocated in empty cages interspersed in the racks [46]. During the paradigm, rats were daily monitored for body weight. For each drinking session, alcohol consumption (g/kg) and preference % over total fluid consumption were measured. Mean alcohol consumption, at 24 and at 1 h after alcohol presentation, was then calculated considering the total number of drinking sessions in each group during pre-conceptional time, gestation, and lactation.

### Behavioral procedures

**Figure 1:**
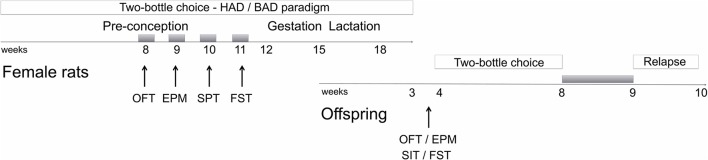
Schematic overview of the experimental design. Gray boxes refer to alcohol deprivation phases. For details, see section Materials and Methods. HAD, Habitual alcohol drinking rats; BAD, Binge alcohol drinking rats; OFT, open field test; EPM, elevated plus maze test; SPT, saccharin preference test; FST, forced swim test; SIT, social interaction test.

Starting from week 8 of the two-bottle choice paradigm, female rats underwent a battery of behavioral assessments that started following 12 h of acute abstinence, during the weekend, and included open field, elevated plus maze, and forced swim tests (Figure 1). Saccharin preference was employed to as a measure of sensitivity to natural rewards, rather the social interaction test, in order to avoid confounding effects due to altered behavioral reactivity as a result of alcohol abstinence.

Male offspring were tested in the open field, elevated plus maze, social interaction, and forced swim test starting from PND 23. Afterwards, they were also tested for alcohol vulnerability, starting from PND 32.

On the test days, the animals were acclimatized for 60 min before the experimental session. The experiments were carried out in a soundproof room between 9:00 and 14:00. Animal performance was recorded and monitored in an adjacent room. The equipment was thoroughly cleaned before animal's entry to ensure that rat's behavior was not affected by the detection of another rat's scent.

### Open field test

Behavioral reactivity in a novel open field arena was measured in a Plexiglas square box (44 cm long, 44 cm wide, and 20 cm high) by employing an automatic video-tracking system (Any Maze, Ugo Basile, Italy), in a mean light (100 lx) illuminated chamber. Each experimental session lasted 5 min [25]. The test produces a qualitative mapping of the motor pattern, measuring total distance traveled (TDT), as a measure of locomotor activity, number of transitions (NTC) from peripheral to central squares of the arena, and amount of time spent on the central areas (ATC) as measures of explorative behavior.

### Elevated plus maze test

Unconditioned anxiety-like behavior was assessed in the elevated plus maze (EPM). EPM apparatus consisted of a plus-shaped platform from dark-gray PVC elevated to a height of 70 cm above the floor. Two of the opposing arms (50 × 10 cm) were closed by 40 cm-high side end-walls (closed arms), whereas the two other arms had no walls (open arms). The arms were oriented perpendicular to each other and connected by an open central area (10 × 10 cm). At the beginning of each session, rats were placed in the center of the maze facing one of the open arms. Time spent on each arm and number of entries were recorded by using ANY MAZE Video Tracking System (Ugo Basile, Italy) along the 5 min test duration. An entry was scored when all the four paws entered into each single arm. The percentage of time spent on the open arms/total time spent on open and closed arms, and of number of entries in the open arms/number of entries in the open and closed arms were analyzed, as they constituted the primary indices of trait anxiety-like behavior in rodents. Information on general activity was obtained by measuring closed arm and total entries [26].

### Saccharin preference test

Anhedonia was assessed using the saccharin preference test [27].

Briefly, during the habituation phase, each cage was supplied with two drinking bottles filled with water to avoid place preference. On the next day, a bottle of water was replaced by one containing 0.2% (w/v) saccharin solution (Sigma-Aldrich, Italy). Saccharin- and water intake were measured by weighing the bottles 24 h later. The location of the two bottles was randomly alternated to avoid location preference bias. Saccharin preference was calculated as percentage of the volume of the saccharin solution consumed divided by the total fluid volume (water plus saccharin).

### Forced swim test

Porsolt test was conducted as already described in Plescia et al. [47]. Briefly, each rat was placed individually in a glass cylinder (40 cm high, 18 cm inside diameter) containing ≈5 l of clean water. Water temperature was maintained at 22–23°C. Rats were forced to swim for 15 min (pre-test). Animals were then allowed to return to their home cages. On the next day, each rat was placed into the water and forced to swim for 5 min (test). The session was videotaped and the duration of immobility, swimming and climbing was recorded (sec). The rat was considered as immobile when it stopped struggling and remained floating in the water, keeping its head above the water. Climbing was defined by active attempts to climb the walls of the cylinder to escape.

### Social interaction test

Social reward was measured by the social interaction test, adapted from Christoffel et al. [48]. The offspring was placed into a novel arena equipped with a small animal cage (10 × 10 cm) at one end. Rat movements were monitored for 5 min in the absence of a social stimulus into the cage (used to determine baseline exploratory behavior), followed by 5 min in the presence of a caged conspecific male rat, matched for age. Partners had not been socially isolated prior to testing and were unfamiliar with both the apparatus and the experimental animal with which they were paired for testing. We measured duration of time spent in the interaction zone, including 2 cm^2^ surrounding the small animal cage, by using Anymaze software (Ugo Basile, Italy). Social interaction was calculated as a ratio between the time spent on the interaction zone when the stimulus rat was present, over the time spent when the stimulus rat was absent.

### Alcohol vulnerability in the offspring

Male offspring underwent home cage two-bottle “alcohol vs. water” choice regimen, and were given 24 h free choice between one bottle of alcohol (10% v/v) and one of tap water, 7 days per week, for 4 weeks, followed by a relapse-like week, after deprivation. Alcoholic solution (10% v/v) was daily prepared by diluting alcohol 96° (Carlo Erba Reagenti, Italy) with tap water. Plastic bottles (120 ml; metal cap 0.8 mm diameter hole, Tecniplast, Italy) were refilled with 100 ml fresh solution every day, and presented at lights off in alternative left-right position, to avoid side preference. Alcohol- and water intake were measured by weighing the bottles. Possible fluid spillage was monitored by using multiple bottles filled with water and alcohol 10%, positioned in empty cages interspersed in the cage racks. During the paradigm, rats were daily monitored for body weight.

### Statistical analysis

The analysis included results from rat dams whose pups were assessed along different phases of the experimental design. The differences in mean alcohol consumption and preference of female rats were analyzed by Student's *t*-test or Mann–Whitney test, when appropriate. The analysis of drinking trajectories of female rats, starting from the last 3 weeks of pre-conceptional time to the end of the drinking paradigm, was performed by two-way ANOVA for repeated measures (RM 2-way ANOVA), followed by Bonferroni *post-hoc* test, on both mean alcohol consumption per week, including “alcohol drinking pattern” as the between-subject factor and “week” as the within-subject factor, and mean weekly alcohol consumption during pre-conceptional time, gestation and lactation, including “alcohol drinking pattern” as the between-subject factor and “period” as the repeated measure factor. Data analysis on behavioral experiments was performed by one-way ANOVA, including “treatment” as the between subjects factor. Data analysis on alcohol vulnerability experiments was performed by RM 2-way ANOVA, including “maternal alcohol drinking pattern” as the between-subject factor and “week” as the within-subject factor. Bonferroni *post-hoc* test was employed, when necessary. Kruskal–Wallis test, followed by Dunn's multiple comparison test, was employed for the analysis of alcohol deprivation effect. Data are reported as mean ± SEM. Statistical significance was set at *p* < 0.05 (α = 0.05).

## Results

### Pre-conceptional and peri-gestational alcohol drinking patterns in female rats

Female rats had habitual—continuous—or binge—intermittent—access to the “two-bottle choice” paradigm during 12 weeks, before mating, during pregnancy and lactation. Fluid spillage, constantly monitored by using multiple bottles filled with water and alcohol 20%, allocated in empty cages interspersed in the racks, was neither relevant nor systematic. Measures of alcohol intake along the paradigm are reported in Table 1. As expected pre-conceptional time BAD paradigm induced higher means alcohol consumption and preference, at both time points considered, 1 and 24 h after alcohol presentation, with respect to HAD. After fecundation, as well as during post-partum days, BAD rats increase in mean alcohol consumption and preference was attenuated with respect to HAD rats.

**Table 1:** Alcohol drinking pattern in habitual alcohol drinking (HAD) rats and binge alcohol drinking (BAD) rats at pre-conceptional, gestational, and post-gestational time.

		**Pre-conception**		**Gestation**		**Post-gestation**	
		**HAD**	**BAD**	**HAD**	**BAD**	**HAD**	**BAD**
Body weight (g)		260.8 ± 2.2	249.5 ± 4.3^*^	316.0 ± 3.6	303.1 ± 6.2	272.5 ± 6.2	267.3 ± 4.1
Mean alcohol consumption (g/kg)	1 h	0.77 ± 0.1	3.4 ± 0.1^◦◦◦^	2.5 ± 0.2	2.4 ± 0.2	3.5 ± 0.5	3.5 ± 0.4
	24 h	3.5 ± 0.1	8.1 ± 0.3^◦◦◦^	3.4 ± 0.4	5.4 ± 0.6^*^	5.6 ± 0.6	8.5 ± 0.4^**^
Alcohol preference %	1 h	52.0 ± 0.3	65.3 ± 0.8^◦◦◦^	55.8 ± 1.8	65.0 ± 2.2^**^	47.9 ± 3.8	55.0 ± 3.5
	24 h	38.8 ± 0.2	54.8 ± 1.1^◦◦◦^	40.1 ± 2.7	48.4 ± 2.4^*^	40.8 ± 4.2	49.0 ± 2.3

*Data refer to mean ± SEM of alcohol consumption and preference of female rats (n = 8) along the 18 weeks of HAD and BAD paradigms*.

* p < 0.05,

** p < 0.01 Student's t-test;

◦◦◦ *p < 0.001 Mann–Whitney test*.

In order to have a more detailed insight into drinking trajectories, mean alcohol intake of HAD and BAD rats, from the last three pre-conceptional weeks throughout peri-gestational time was analyzed. The RM 2-way ANOVA on mean alcohol consumption during the 9 weeks considered showed a significant effect of alcohol drinking pattern [*F*_(1, 14)_ = 71.07, *p* < 0.0001], weeks [*F*_(8, 112)_ = 7.465, *p* < 0.0001], and their interaction [*F*_(8, 112)_ = 3.453, *p* = 0.0014]. In particular, BAD rats displayed increased alcohol consumption with respect to HAD rats on week 10, 11, and 12 of pre-conceptional period (*t* = 6.091, *p* < 0.001; *t* = 4.099, *p* = 0.0007; *t* = 5.354, *p* < 0.001); during gestational week 3 (*t* = 2.877, *p* = 0.0424) and post-gestational weeks 1 and 3 (*t* = 3.015, *p* = 0.0279; *t* = 4.788, *p* < 0.001).

**Figure 2:**
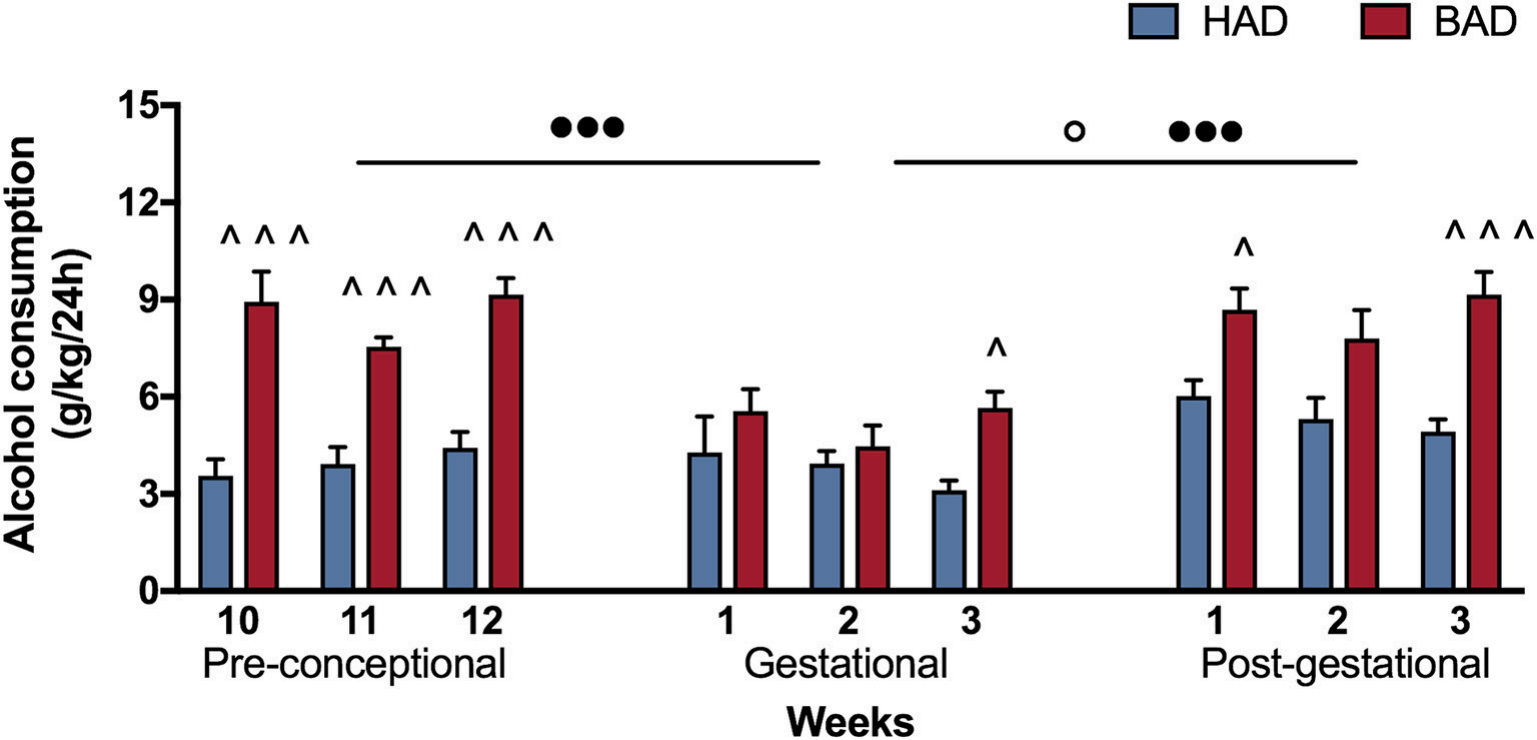
Female rat alcohol drinking trajectories. Binge alcohol drinking (BAD) rats showed higher alcohol consumption than habitual alcohol drinking (HAD) rats during the last three pre-conceptional weeks, gestation and post-gestation. During gestation, BAD rats displayed a significant decrease in alcohol consumption with respect to the last three pre-gestational weeks. During post-partum days, drinking consumption of both groups increased with respect to gestational period, reproducing similar trajectories as recorded on pre-conceptional time. Each bar represents the mean ± SEM of *n* = 8 rats. ^∧^*p* < 0.05, ^∧∧∧^*p* < 0.001 vs. HAD rats; ^◦^*p* < 0.05 within HAD group; ^•••^*p* < 0.001 within BAD group.

Moreover, when we analyzed the effects of the three periods considered, the RM 2-way ANOVA showed a significant effect of period [*F*_(2, 4)_ = 51.53, *p* = 0.0014], alcohol drinking pattern [*F*_(1, 2)_ = 45.05, *p* = 0.0215], and their interaction [*F*_(2, 4)_ = 21.33, *p* = 0.0073]. After fecundation, BAD rats decreased their alcohol consumption with respect to their pre-conceptional levels (*t* = 9.70, *p* = 0.0020). During post-partum days, the drinking trajectories of both groups increased with respect to gestational period (HAD: *t* = 4.688, *p* = 0.0282; BAD: *t* = 9.715, *p* = 0.0019), and reproduced a similar pattern as on pre-conceptional time (Figure 2).

### Behavioral assessment in female rats

#### Open field test

**Figure 3:**
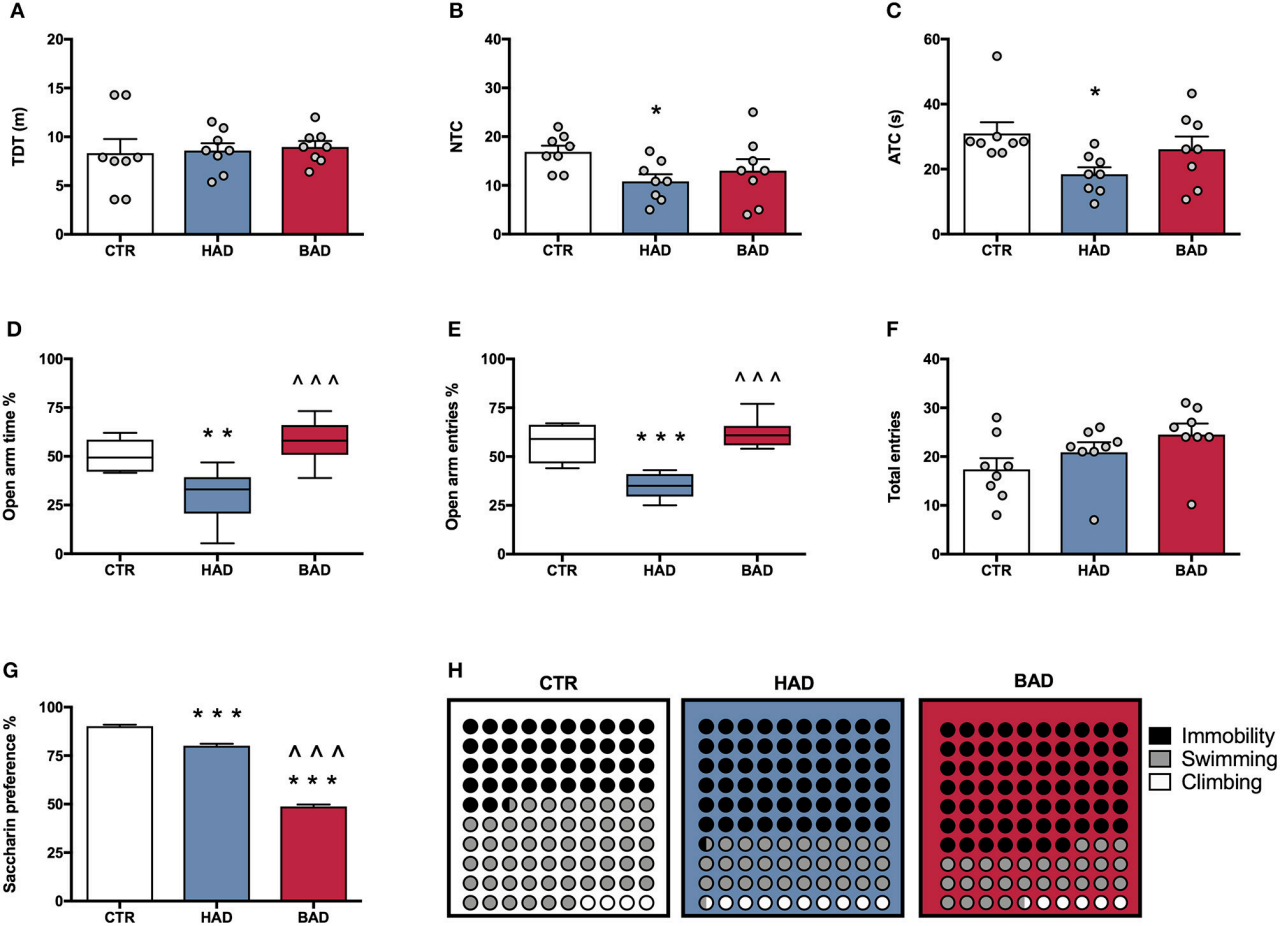
Female rat pre-conceptional phenotype. Habitual (HAD) and binge (BAD) alcohol drinking patterns affect behavioral reactivity, anxiety-like behavior, reward sensitivity, and depression-like behavior. Open field test—**(A)** total distance traveled (TDT); **(B)** number of transitions in central areas (NTC); and **(C)** amount of time spent on the central areas of the arena (ATC). Elevated plus maze test—**(D)** percentage of time spent on the open arm; **(E)** percentage of entries in the open arm; **(F)** total entries. **(G)** Saccharin preference; **(H)** Forced swim test, where the parts-of-whole−10 × 10 dot—plots represent normalized mean values from immobility time (black); swimming time (gray); climbing time (white) in the three experimental groups. Each bar represents the mean ± SEM of *n* = 8 rats. Each box-and-whisker plot represents the median (horizontal line in the box), 25–75% (box) and min-to-max (whiskers) values of *n* = 8 rats. *p < 0.05, **p < 0.01; ***p < 0.001 vs. CTR rats; ^∧∧∧^*p* < 0.001 vs. HAD rats.

At the end of week 8, female rats' performance in the open field was assessed as a measure of locomotor activity and behavioral reactivity in a novel environment, following 12 h alcohol deprivation. Results from a one-way ANOVA showed no significant differences in locomotor activity, in terms of TDT, among the three experimental groups [*F*_(2, 21)_ = 0.1032, *p* = 0.9024; Figure 3A]. Statistical analysis indicated significant differences in NTC [*F*_(2, 21)_ = 3.895, *p* = 0.0364], and ATC [*F*_(2, 21)_ = 4.516, *p* = 0.0234]. In particular, Bonferroni *post-hoc* test highlighted a significant decrease in both NTC (*t* = 2.721, *p* < 0.05) and ATC (*t* = 3.005, *p* < 0.05) in HAD- with respect to CTR rats (Figures 3B,C).

#### Elevated plus maze test

At the end of week 9, female rats were tested in the EPM to evaluate anxiety-like behavior at 12 h alcohol deprivation. One-way ANOVA, performed on data from the percentage of time spent on open arm/time on open and closed arms, showed significant differences among the experimental groups [*F*_(2, 21)_ = 14.42, *p* = 0.0001]. In particular, Bonferroni *post-hoc* test indicated a significant lower preference for the open arms in HAD rats, compared to CTR (*t* = 3.959, *p* < 0.01), while BAD rats spent more time in the open arms than HAD group (*t* = 5.121, *p* < 0.001; Figure 3D). When the percentage of open arm entries out of total entries was analyzed, one-way ANOVA showed significant differences among the three experimental groups [*F*_(2, 21)_ = 26.13, *p* < 0.0001]. In particular, Bonferroni *post-hoc* analysis showed a significant decrease in HAD rats, with respect to CTR (*t* = 5.604, *p* < 0.001), and an increase in BAD-, with respect to HAD rats (*t* = 6.758, *p* < 0.001; Figure 3E). No significant differences among the three experimental groups in number of total entries were observed [*F*_(2, 21)_ = 2.579, *p* = 0.0997; Figure 3F).

#### Saccharin preference test

At the end of week 10, female rats underwent 12 h of alcohol deprivation and were subjected to saccharin preference test, in order to evaluate their response to a natural reward during abstinence. Results from one-way ANOVA showed significant differences among the three experimental groups [*F*_(2, 21)_ = 581.0, *p* < 0.001]. In details, Bonferroni *post-hoc* test highlighted a significant decrease in saccharin preference in both HAD-(*t* = 7.961, *p* < 0.001) and BAD rats (*t* = 32.71, *p* < 0.001), with respect to CTR. Moreover, BAD rats displayed a significant decrease in saccharin preference when compared to HAD rats (*t* = 24.74, *p* < 0.001; Figure 3G).

#### Forced swim test

The experimental groups were evaluated for depressive-like behavior in the Porsolt test at the end of week 11. After 12 h of alcohol abstinence, rats were exposed to the pretest and, at 36 h abstinence, rats underwent the 5-min Test session, when immobility, swimming, and climbing time were recorded. One-way ANOVA showed a significant effect of treatment on immobility [*F*_(2, 21)_ = 30.92, *p* < 0.0001]. Bonferroni *post-hoc* test highlighted a significant increase both in HAD (*t* = 6.441, *p* < 0.001) and in BAD rats (*t* = 7.127, *p* < 0.001) with respect to CTR. In line with these results, a significant effect of treatment was observed on swimming time [*F*_(2, 21)_ = 15.36, *p* < 0.001], with a significant decrease in both HAD- (*t* = 4.375, *p* < 0.001) and BAD-rats (*t* = 4.019, *p* < 0.01), when compared to controls. Furthermore, when the parameter climbing was analyzed, significant differences were observed among groups [*F*_(2, 21)_ = 10.87, *p* = 0.0006]: HAD rats displayed a significant increase in climbing with respect to CTR (*t* = 4.491, *p* < 0.001), and BAD-rats (*t* = 3.331, *p* < 0.01). Figure 3H shows the composite behavioral pattern of the three experimental groups.

### Behavioral assessment in the offspring

#### Open field test

**Figure 4:**
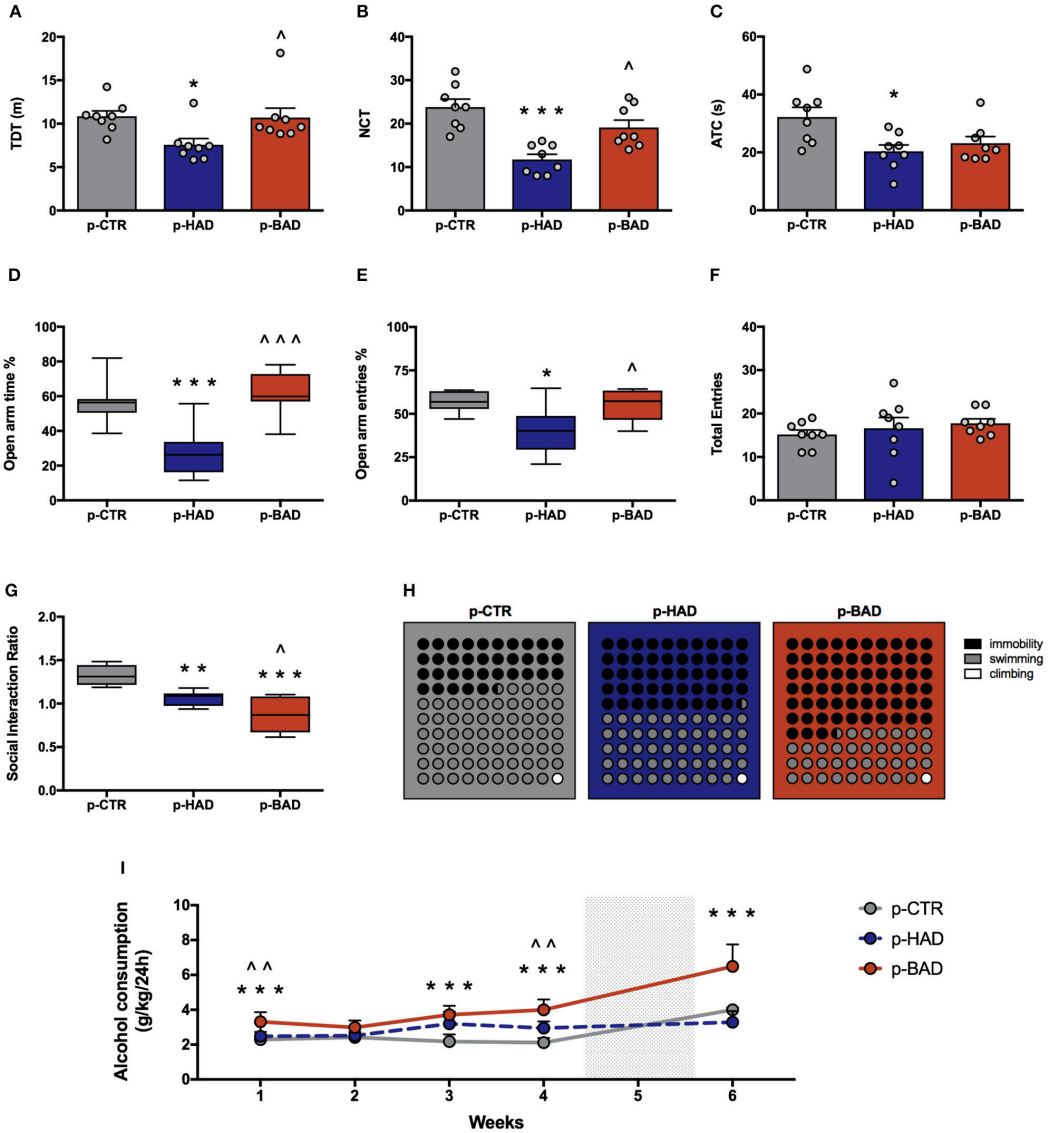
Offspring phenotype and alcohol vulnerability. Pre-and post-gestational habitual (p-HAD) and binge (p-BAD) alcohol drinking affect offspring behavioral reactivity, anxiety-like behavior, social reward sensitivity, and depression-like behavior. Open field test—**(A)** total distance traveled (TDT); **(B)** number of transitions in central areas (NTC); and **(C)** amount of time spent on the central areas of the arena (ATC). Elevated plus maze test—**(D)** percentage of time spent on the open arm; **(E)** percentage of entries in the open arm; **(F)** total entries. **(G)** Social interaction test; **(H)** Forced swim test, where the parts-of-whole−10 × 10 dot—plots represent normalized mean values from immobility time (black); swimming time (gray); climbing time (white) in the three experimental groups. **(I)** Vulnerability to alcohol abuse is measured in the two bottle choice paradigm during drinking induction (weeks 1–4) and relapse following forced abstinence (week 6). Each bar and each dot represents the mean ± SEM of *n* = 8 rats. Each box-and-whisker plot represents the median (horizontal line in the box), 25–75% (box) and min-to-max (whiskers) values of *n* = 8 rats. *p < 0.05, **p < 0.01; ***p < 0.001 vs. p-CTR rats; ^∧^*p* < 0.05; ^∧∧^*p* < 0.01; ^∧∧∧^*p* < 0.001 vs. p-HAD rats.

Offspring's performance in the open field was assessed as a measure of locomotor activity and behavioral reactivity in a novel environment. One-way ANOVA showed significant effects of maternal alcohol drinking pattern on locomotor activity, in terms of TDT, among the three experimental groups [*F*_(2, 21)_ = 4.915, *p* = 0.0177]; indeed, p-HAD offspring showed decreased locomotor activity, with respect to both p-CTR (*t* = 2.776, *p* < 0.05) and p-BAD offspring (*t* = 2.65, *p* < 0.05; Figure 4A). Moreover, significant differences in NTC were observed [*F*_(2, 21)_ = 14.8, *p* < 0.001], with a decrease in p-HAD when compared to p-CTR (*t* = 5.396, *p* < 0.001) and p-BAD offspring (*t* = 3.293, *p* < 0.05).

Analysis of ATC data also showed significant differences [*F*_(2, 21)_ = 5.441, *p* = 0.0125]. In particular, Bonferroni *post-hoc* test highlighted a significant decrease in p-HAD offspring with respect to p-CTR rats (*t* = 3.155, *p* < 0.05; Figures 4B,C).

#### Elevated plus maze test

Animals were tested in the EPM to evaluate anxiety-like behavior. One-way ANOVA, performed on data from the percentage of time spent on open arm/time on open and closed arms, showed significant differences among the experimental groups [*F*_(2, 21)_ = 16.05, *p* < 0.0001]. In particular, Bonferroni *post-hoc* test indicated a significant lower preference for the open arms in p-HAD offspring, compared to p-CTR (*t* = 4.466, *p* < 0.001), while p-BAD offspring spent more time in the open arms than p-HAD ones (*t* = 5.253, *p* < 0.001; Figure 4D). When the percentage of open arm entries out of total entries was analyzed, one-way ANOVA showed significant differences among the three experimental groups [*F*_(2, 21)_ = 6.559, *p* = 0.0061]. In particular, Bonferroni *post-hoc* analysis showed a significant decrease in p-HAD offspring, with respect to p-CTR (*t* = 3.284, *p* < 0.05), and an increase in p-BAD, with respect to p-HAD offspring (*t* = 2.965, *p* < 0.05; Figure 4E). No significant differences among the three experimental groups in number of total entries were observed [*F*_(2, 21)_ = 0.6072, *p* = 0.5542; Figure 4F].

#### Social interaction test

Offspring was subjected to the social interaction test in order to evaluate their response to social reward. Results from one-way ANOVA showed significant effects of maternal alcohol drinking pattern among the three experimental groups [*F*_(2, 21)_ = 21.51, *p* < 0.001]. In details, Bonferroni *post-hoc* test highlighted a significant decrease in social interaction in p-BAD, which showed the lowest values (*t* = 2.871, *p* < 0.05), and p-HAD offspring, with respect to p-CTR (*t* = 6.542, *p* < 0.001; *t* = 3.671, *p* < 0.01; Figure 4G).

#### Forced swim test

The experimental groups were evaluated for depressive-like behavior in the Porsolt test. Offspring underwent a 5-min test session 24 h after the pretest session, when immobility, swimming, and climbing time were recorded. One-way ANOVA showed a significant effect of treatment on immobility [*F*_(2, 21)_ = 30.08, *p* < 0.0001]. Bonferroni *post-hoc* test highlighted a significant increase both in p-HAD- (*t* = 3.978, *p* < 0.01) and in p-BAD offspring (*t* = 7.756, *p* < 0.001) with respect to p-CTR. Moreover, p-BAD offspring displayed a significant increase in immobility time when compared to p-HAD offspring (*t* = 3.778, *p* < 0.01).

In agreement with these results, a significant effect of treatment was observed on swimming time [*F*_(2, 21)_ = 34.54, *p* < 0.001], with a significant decrease both in p-HAD- (*t* = 4.241, *p* < 0.01) and in p-BAD offspring (*t* = 8.311, *p* < 0.001), when compared to p-CTR. Again, p-BAD offspring displayed a significant decrease in swimming time when compared to p-HAD offspring (*t* = 4.069, *p* = 0.0017).

When the parameter climbing was analyzed, no significant differences were observed [*F*_(2, 21)_ = 0.391, *p* = 0.6809]. Figure 4H shows the composite behavioral pattern of the three experimental groups.

#### Alcohol vulnerability

Offspring had access to the “two-bottle choice” paradigm from PND 32 for 4 weeks, followed by a relapse-like week, after deprivation in order to test them for alcohol deprivation effect. RM 2-way ANOVA on mean alcohol intake (g/kg) along the first 4 weeks of the paradigm showed a significant effect of maternal alcohol drinking pattern [*F*_(2, 21)_ = 36.73, *p* < 0.0001], weeks [*F*_(3, 63)_ = 4.856, *p* = 0.0042], and their interaction [*F*_(6, 63)_ = 3.825, *p* = 0.0026]. In particular, offspring from BAD dams showed higher alcohol intake with respect to p-CTR on week 1, 3, and 4 (*t* = 4.091, *p* = 0.0003; *t* = 6.164, *p* < 0.0001; *t* = 7.548, *p* < 0.0001), and with respect to p-HAD offspring on week 1 and 4 (*t* = 3.325, *p* = 0.0039; *t* = 4.192, *p* = 0.0002). Moreover, p-HAD offspring showed increased alcohol intake with respect to p-CTR rats on week 3 and 4 (*t* = 4.096, *p* = 0.0003; *t* = 3.356, *p* = 0.0036). When alcohol deprivation effect was measured as mean alcohol intake (g/kg) during the relapse-like time window, following a week of alcohol abstinence, Kruskal–Wallis test showed a significant effect of maternal alcohol drinking pattern (*p* < 0.001). In details, Dunn's multiple comparison test showed an increase in alcohol consumption in p-BAD- when compared with p-HAD-offspring (*p* < 0.01) and p-CTR ones (*p* = 0.0295; Figure 4I).

## Discussion

The present study aimed at evaluating the consequences of pre-conceptional long-term binge-like alcohol self-administration on different behavioral dimensions in female rats; moreover, we prolonged alcohol exposure during pregnancy and lactation in order to evaluate the occurrence of relevant effects on adolescent male offspring's phenotype, including the inheritance of alcohol abuse. Our results show that the experimental model of BAD provides a robust opportunity to explore the discrete consequences of this worldwide spread, excessive alcohol drinking habit, and highlight the occurrence of the inheritance of mood- and reward-related disturbances in the offspring.

Indeed, we modeled binge drinking in female rats by using a two-bottle choice paradigm with water and intermittent access to 20% alcohol, over 12 weeks. In order to explore discrete pattern-related effects, a continuous/habitual alcohol-drinking group was included, as positive control. In accordance with previous reports in both male and female rats [21, 46, 49–51], the intermittent access to alcohol reliably models excessive binge-like alcohol consumption. Indeed, BAD rats showed a significantly higher alcohol intake than HAD rats [52]: within the first hour of alcohol access they drank intoxicating amounts of alcohol [46], in accordance with the definition of binge drinking [53]. The “limited-access” model appropriately recreates the binging patterns observed in some human alcoholics [53–55] and, importantly, the voluntary self-administration prevents rats from undergoing stressful procedures that may contribute to the alteration of the subsequent behavioral outcomes. Indeed, once rats acquired a steady drinking behavior, a characterization of the affective phenotype was carried out on alcohol deprivation days.

Our data show that alcohol-drinking pattern is a determiner in the discrete modifications of the emotional and affective homeostasis in female rats here observed. Indeed, whether in the open field both HAD- and BAD female rats did not differ from CTR in terms of locomotor activity, HAD rats showed a significant avoidance of the central zone of the arena, while BAD rats did not differ from controls. This finding was confirmed by the results of the elevated-plus maze, where only HAD rats displayed increased anxiety-like behavior. Notably, the intermittent pattern of alcohol consumption is not associated to overt typical signs of alcohol withdrawal: mainly, rats trained in the intermittent model display cognitive deficits rather than increased anxiety-like behavior during acute abstinence [56, 57]. However, no significant differences appeared in the number of total arm entries, ruling out any impairment in locomotor activity [58].

To further analyze the consequences of binge-like alcohol consumption, anhedonia, and depression-like behavior were assessed. Anhedonia, i.e., the decreased experience of pleasure in response to nondrug reward [59], is a key symptom of depressive disorders, commonly described in subjects who are long-term abstinent from drugs of abuse: the development of a negative effect, such as anhedonia associated to dysphoria, is a relevant vulnerability factor for relapse (DSM-V, [60, 61]). In the saccharin preference test we evaluated animals' preference between regular water and a sweet solution, which represents a highly rewarding natural stimulus for rodents. Interestingly, whether habitual alcohol exposure induced a lower preference for the natural reward with respect to CTR, the most anhedonic response was observed in BAD rats which showed significant lower preference for saccharin than HAD rats. The reduction in the affective tone was confirmed by the results of the forced swim test, where BAD rats showed longer immobility score, when compared both to CTR and HAD rats, suggesting the occurrence of “despair-like behavior” as a consequence of the binge-like drinking exposure. On the other hand, HAD rats displayed a less defined picture, in that they showed a lower immobility time with respect to BAD rats and an increase in climbing, which suggests the expression of lower behavioral despair [62].

Overall, binge-like alcohol drinking seems to be responsible for a depressive-oriented phenotype, when animals are tested on abstinence days, which includes anhedonia- and despair-, whereas HAD rather induces an anxiety-oriented behavior. The repeated cycles of alcohol exposure and withdrawal typical of binge-like drinking may lead to enduring aberrant plasticity in strategic brain circuitries that drive a transition from positive to negative reinforcement-based alcohol seeking, and abstinence-induced affective disturbances [57, 63–74]. Supplementary and in depth evidence on the subject are available on seminal reviews [75].

Notably, after fecundation, alcohol consumption was evidently modified and differences in drinking trajectories were attenuated during pregnancy and lactation. This observation is in agreement with data showing that in the general female population the reproductive state is able to modulate alcohol intake and preference [76]. In accordance with our previous reports (51), during post-partum days, the drinking patterns reproduced similar trajectories as recorded on pre-conceptional time. The following aim of this research was the investigation of the consequences of perinatal alcohol exposure on the behavioral phenotype of the offspring. Few studies have addressed the multigenerational effects of maternal binge drinking; among them, a recent research demonstrated that maternal binge drinking induces a subtle range of cognitive defects, mainly referred to working memory deficits, associated with persistent neuroinflammation and myelin damage [77]. The present research widens the extent of the detrimental consequences of maternal binge drinking to the affective dimension: indeed, maternal binge-like alcohol drinking resulted in the inheritance of a depression-like phenotype in the offspring, when tested in adolescence. In details, whereas p-HAD offspring showed anxiety-like behavior in the open field and in the EPM, a dramatic decrease in social reward sensitivity in the social interaction, and a higher despair-like behavior in the FST characterized p-BAD progeny as adolescents. As a matter of fact, p-HAD progeny displayed the same alterations in coping with inescapable stress as their mothers, and a higher social interaction ratio than p-BAD offspring but lower than p-CTR.

Social interaction, which allows studying social attitudes in the experimental model, is experienced as a rewarding event in animals as in humans [78, 79]. In particular, whereas an age-typical inhibition of social behavior occurs in adulthood [80], social interactions are considered highly rewarding in young mammals [28]. Recent neuroimaging studies have shown that the activation of the reward system mediates the processing of social stimuli in a similar manner as nonsocial rewards and thus motivates social behavior [81]. Early experiences, such as prenatal alcohol exposure, may influence the ongoing development of reward-related circuitries (reviewed in 82, 83). The evidence that the major disruption in social behavior occurred in BAD progeny is not easy to explain. We can hypothesize that the long-term binge-like alcohol drinking affects the physiological response to natural rewarding stimuli in the mothers and that the inheritance of such disturbance may alter future social behavior in the offspring. In accordance, a longitudinal prospective study showed that pre-gestational exposure to alcohol binge drinking may be a risk factor for decreased social interaction, attention, hyperactivity in childhood [84], and is associated with substance dependence or abuse disorders vulnerability in early adulthood [34]. Consistently, our data show, for the first time, a specific vulnerability to alcohol abuse in the offspring of BAD dams. Indeed, when all groups were exposed to the two-bottle choice with alcohol solution and water, from adolescence to early adulthood, p-BAD progeny displayed a relevant increase in alcohol consumption compared to p-HAD- and CTR progeny along the paradigm, which suggests a faster acceptance and a higher preference for alcohol. Moreover, p-BAD progeny displayed a significant increase in voluntary alcohol consumption after a week of forced abstinence, resembling the so called “alcohol deprivation effect” (ADE). This phenomenon is well-established as a robust and reliable measure of the motivation to seek and consume alcohol, loss of control, and relapse in rats [85]. The evidence of ADE only in p-BAD offspring is suggestive of the occurrence of peculiar signs of vulnerability to alcohol abuse, such as compulsive alcohol-seeking and reinstatement behavior. Unfortunately, the time window for ADE assessment had to be short in order to study the phenomenon from adolescence to early adulthood. This allowed us to parallel clinical evidence of equivalent 14–25 years human subjects, along the temporal window when maturation of cortical and limbic regions is under completion [86]. A longer exposure to the drinking procedure and/or to the operant chamber will help to further investigate the reward-related responses to alcohol of p-BAD progeny, in the next future.

Overall, the idea that the progeny can epigenetically inherit aberrant functions due to parental alcohol abuse is not a novel issue. Recently, pre-conceptional alcohol intake has been reported to negatively affect offspring adult health by increasing stress hormone response to an immune challenge and altering the expression and methylation profiles of stress regulatory genes in various brain areas: this evidence suggests that pre-conceptional alcohol intake may play a role in the inheritance of stress-related diseases [87]. Moreover, clinical and epidemiological studies indicate that pre-conceptional exposure to alcohol is not only associated with the risk for alcohol abuse in adolescent and young adult humans [88, 89], but is perhaps the best predictor of later alcohol abuse [90, 91].

In conclusion, this study shows that pre-conceptional and peri-gestational habitual and binge-like alcohol consumption induce respectively an anxiety-like and a depression-like oriented phenotype in the mothers, which can be accordingly inherited by the adolescent male progeny. These preclinical findings prompt the early detection of discrete psychopathological traits that may represent predictive signs of alcohol vulnerability in the adolescents, in order to carry out rescue strategies for at risk endophenotypes [92–98]. The occurrence of similar inheritance in the female progeny is still to be ascertained. Ultimately, our findings highlight the need for more incisive public educational campaigns that can educate women of childbearing age avoiding binge—and continuous—alcohol drinking during pregnancy, in order to increase their awareness on the severe consequences they may expose their offspring, even at a later developmental epoch.

## Author contributions

AB experimental procedures, contribution to experimental design, and writing. FP statistical analysis and graphical layout. VC, AC, GL experimental procedures, contribution to writing. CC experimental design, data interpretation, and writing. All of the authors read and approved the final manuscript.

### Conflict of interest statement

The authors declare that the research was conducted in the absence of any commercial or financial relationships that could be construed as a potential conflict of interest.
